# Spermless males fail to remedy the low fecundity of parthenogenetic females in *D. melanogaster*

**DOI:** 10.17912/micropub.biology.001446

**Published:** 2025-02-04

**Authors:** Lewis I. Held, Jr., Surya J. Banerjee, Dylan W. Schwilk, Souvik Roy, Kambre A. Huddleston, Jason J. Shin

**Affiliations:** 1 Dept. of Biological Sciences, Texas Tech University, Lubbock, Texas, United States

## Abstract

The recent construction of a parthenogenetic strain of *D. melanogaster* offers new avenues of research, but this potential is limited by the stock’s abysmal fecundity. We tried using spermless (placebo) males to “trick” the virgins into producing more offspring, but the boost that we achieved proved to be short-lived due to premature death of the mothers. To explore the cause of this mortality, we compared the lifespans of parthenogenetic vs. wild-type females when mated with spermless males. We found that parthenogenetic females are less robust than wild-females when raised alone but are more resistant to harm from spermless males.

**Figure 1. Effects of spermless males on fecundity (a) and longevity (b-d) of parthenogenetic (a-c) or wild-type (d) females:**
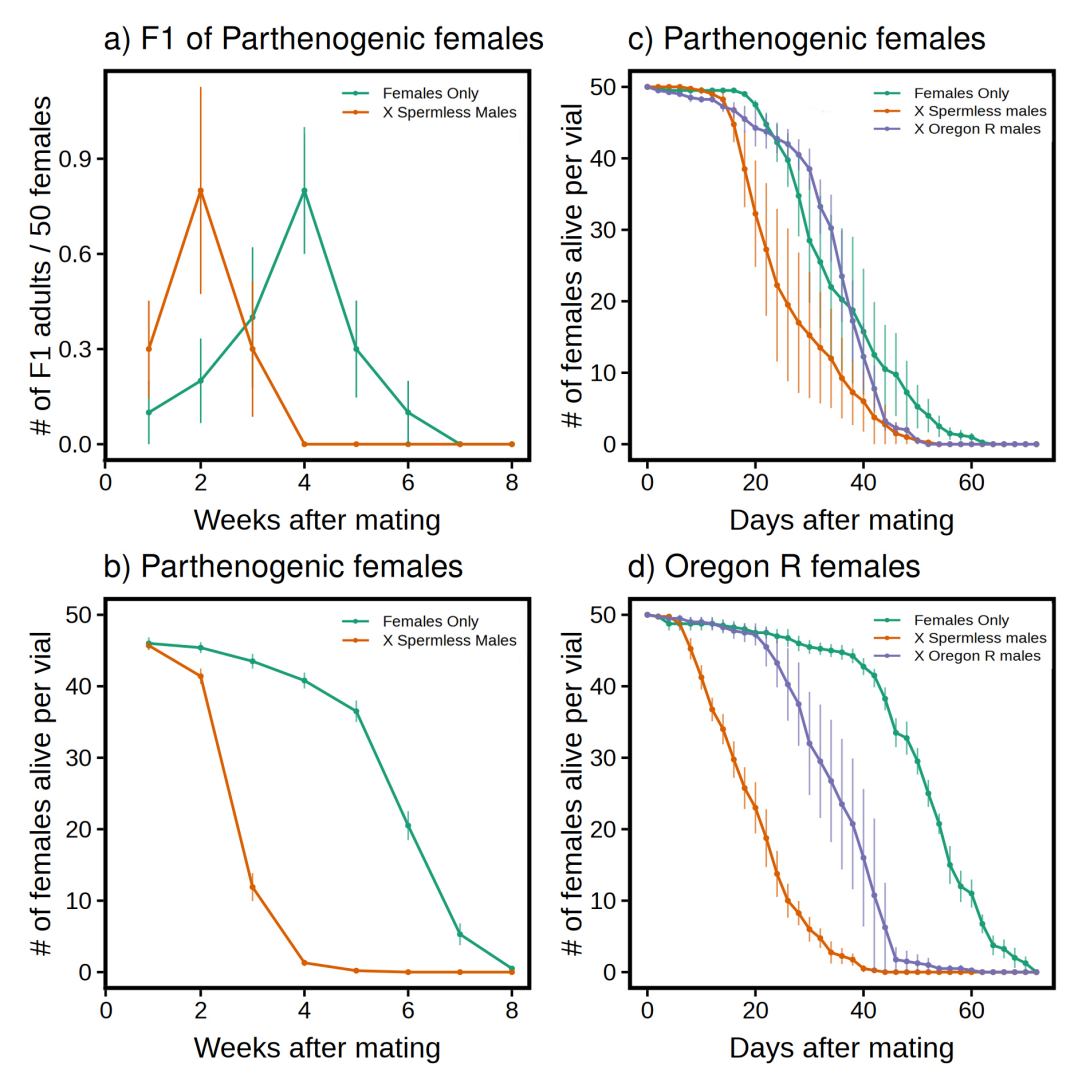
Graphs show mean values over time across vials (n=10 for a and b; n=4 for c and d), and vertical lines show standard errors.

## Description

Sperling et al. (2023) succeeded in constructing a parthenogenetic strain of *D. melanogaster* by inserting genes from a distantly related species where females naturally reproduce without males. Those genes prod the egg’s nucleus to compensate for the lack of a sperm nucleus by fusing with 1, 2, or 3 “polar bodies” left over from meiosis, leading to diploid (2n), triploid (3n), or tetraploid (4n) offspring, respectively. In this way, the egg circumvents the embryonic lethality associated with a haploid (1n) complement of chromosomes (Santamaria, 1983) .

We have been interested in obtaining 4n flies because their huge cells could help us to solve longstanding puzzles about how cuticular patterning occurs at the cellular level (Held, 1979) . Unfortunately, the frequency of 4n F _1_ from unmated transgenic females is too low to be practical (Sperling et al. only got 143 viable F _1_ from 21,406 virgins!), so we sought ways to increase the fecundity of the virgins. Xue and Noll (2000) had shown that wild-type flies can be spurred to boost their egg production when crossed with sterile males, so we tried using this strategy with the parthenogenetic flies. Here we report difficulties that we encountered with that approach.

Initially, this “placebo” trick seemed to work. Virgins had twice as many offspring within two weeks after males were added ( Fig. 1 a, mean age to peak F1 was 2.0 vs 3.7 weeks, p = 0.0063), but the burst faded quickly as mothers began to die ( Fig. 1 b, significantly different survivorship curves, p < 0.0001), and the overall yield of F _1_ flies (14) failed to exceed the total from unmated females (19). Why were the females dying? We entertained two possible explanations: (1) the transgenic flies are too weak to sustain higher rates of egg-laying, or (2) the spermless males are killing them.

Hypothesis 1 predicts that wild-type females should die less quickly than parthenogenetic ones when crossed with spermless males, while Hypothesis 2 predicts that they die at least as fast. Similarly, Hypothesis 1 predicts that wild-type males should have the same lethal effect on parthenogenetic females as spermless males, while Hypothesis 2 predicts no such effect. Our follow-up experiment was designed to test these predictions, using unmated females as controls. We established six cohorts of flies, with 4 vials/cohort and 50 females/vial, for a total of 200 females/cohort. Two cohorts with females without males served as controls, while the other four contained 25 males added per vial:

Cohort 1: *pMD/TM6B* females; no males.

Cohort 2: *pMD/TM6B* females X spermless males.

Cohort 3: *pMD/TM6B* females X Oregon R wild-type males.

Cohort 4: Oregon R wild-type females; no males.

Cohort 5: Oregon R wild-type females X spermless males.

Cohort 6: Oregon R wild-type females X Oregon R wild-type males.

The results of these reciprocal crosses are plotted in Fig. 1, c and d. Treatment (no males, spermless males, or Oregon R males) had a significant effect on survivorship (p < 0.0001) and this effect interacted with female type (p = 0.0029). Wild-type females die faster than parthenogenetic ones when mated with spermless males (midpoint = 18 vs. 25 days), which is consistent with Hypothesis 2, and parthenogenetic females manifest no comparable lethality when mated with wild-type males, which also agrees with our expectations from Hypothesis 2. Although wild-type females appear more robust than parthenogenetic ones based on longevity under celibate conditions ( Fig. 1 c, d), they are actually *less* able to tolerate the toxic effects of spermless males.

As for the nature of the toxicity, previous work has traced the “mate harm” (Verma et al., 2024) that is done to females (Travers et al., 2015) to a key component of the seminal fluid (Chapman, 2008) that spermless males transmit to females during copulation (Rossant et al., 2023)—namely, the so-called “sex peptide” (Wigby and Chapman, 2005) . Spermless males do more harm than wild-type males because females mate with them more often (Xue and Noll, 2000) , thereby making a bad situation worse (Chapman et al., 1995) .

Given this high specificity, it should come as no surprise that knockouts of the receptor for sex peptide can lessen or eliminate the damaging effects of seminal fluid (Amaro et al., 2024) . Hence, the relative immunity of parthenogenetic *pMD/TM6B* females (relative to wild-type females) that we uncovered in our experiments could conceivably be due to a mutation in the gene for that receptor, which may have occurred during the CRISPR procedure that inserted exogenous DNA constructs. This hypothesis seems farfetched, but at least it should be testable.

## Methods

Two different experiments were conducted. In the first one ( Fig. 1 a, b), we crossed spermless ( *tudor ^1^ bw ^1^ speck ^1^* /+) males with virgin parthenogenetic ( *pMD/TM6B* ) females (see Reagents) and monitored (1) the number of offspring developing to the pharate adult stage ( Fig. 1 a) relative to F _1_ from females alone, and (2) the number of females surviving weekly transfers to fresh food ( Fig. 1 b). Parental females (500 total @50/vial) were kept for ~1 week (3-8 days after collection as pharates) before crossing with 25 spermless males/vial. During that week, a few failed to eclose or died after eclosion, hence reducing the totals by the time of mating to slightly below 500 (= 469 or 466 for uncrossed or crossed females, respectively). After transferring flies to fresh vials, the vacated vials were inspected for any mature F _1_ for two weeks. Roughly half the recovered F _1_ died as pharate adults, while the other half eclosed as viable adults.

In our second experiment, we set up 6 cohorts (@4 vials/cohort and 50 females/vial for a total of 200 females/cohort), but in this case we mated the females within less than a week after eclosion (keeping them at 18˚C in the interim). Flies had to be transferred every 2 days (vs. 1 week) because vials with wild-type males (Cohorts 3 & 6) had roiling seas of larvae by day 3 which drowned any parents that landed on them. To avoid any extraneous variables, we treated the other 4 cohorts alike (i.e., transfers every 2 days) even though there was no larval overcrowding. Survival midpoints are indicated by dashed lines in Figure 1 c and 1d. Analogous midpoints for spermless males (not plotted) occur at 51 or 49 days when crossed with parthenogenetic or wild-type females, respectively (= more than twice the longevity of females). Strangely, wild-type males die younger than spermless males: their midpoints occur at 33 or 40, respectively.

Data analyses were conducted in the R programming language version 4.3.3 (R Core Team, 2024) . In our first experiment, we tested if the mean age in weeks from mating to F _1_ maturity differed across treatments (females only vs spermless males) using a mixed Poisson regression with the glmmTMB package in R (Brooks et al., 2017). To test for differing survivorship curves across treatments in both experiments, we fitted mixed effect Cox proportional hazards models with the coxme package in R (Cox, 1972; Therneau, 2024). In the mean age to F1 adult model and in both survivorship models, vial identity was treated as a random effect. Data and software code are available on github (https://github.com/schwilklab/drosophila) and are archived at https://doi.org/10.5281/zenodo.14775842.

## Reagents

Aside from our wild-type (Oregon R) stock, two mutant strains of flies were used: (1) a “ *tudor* ” stock ( *tudor ^1^ bw ^1^ speck ^1^* / *CyO* ) obtained from the Bloomington Stock Center (#1786) and (2) a “ *pMD/TM6B* ” parthenogenetic stock ( *GFP-polo* ^+^ ; *Myc* ^dp^ *Desat2 ^-^* / *TM6B, Tubby ^1^* ) that was constructed by Sperling et al. (2023) and sent to the lead author (LIH) by Sperling's coauthor, David Glover (CalTech). *Myc* ^dp^ is a duplication of the fly's endogenous *Myc* gene, also known as *diminutive* (Kappes et al., 2011) , while *Desat2 ^-^* is a hypomorphic allele of the *Desaturase2* gene--a natural variant that is found in most populations (Greenberg et al., 2003) . Both of these mutant alleles are explained in Sperling et al. (2023). Only heterozygous virgins from the *pMD/TM6B* stock were used because *pMD* homozygotes exhibit higher mortality and lower fertility. Spermless males were the sons of brown-eyed, non-Curly *tudor* mothers crossed with Oregon R wild-type males. Flies were sexed prior to eclosing from the pupal case. *Drosophila* Instant Medium, (Carolina Biological Supply Blue Formula 4-24) was added to 1-inch diameter plastic vials plus enough tap water (with anti-fungal Tegosept) to form a 1-inch high gel in each vial. Fleischmann’s live yeast grains were added atop the gel to cover ~1/4 of the surface and then wetted with tap water. It was critical to re-wet the food every day (with a syringe) to prevent flies from dying of thirst. All flies were kept at 25˚C.
